# Test Plan for the Verification of the Robustness of Sensors and Automotive Electronic Products Using Scenario-Based Noise Deployment (SND)

**DOI:** 10.3390/s21103359

**Published:** 2021-05-12

**Authors:** Laszlo Heinold, Agnes Barkanyi, Janos Abonyi

**Affiliations:** 1 Continental AG, 8200 Veszprém, Hungary; Laszlo.Heinold@continental-corporation.com; 2 Department of Process Engineering, University of Pannonia, 8200 Veszprém, Hungary; barkanyia@fmt.uni-pannon.hu; 3 MTA-PE Lendület Complex Systems Monitoring Research Group, University of Pannonia, 8200 Veszprém, Hungary

**Keywords:** sensor development, robustness, noise deployment, quality function deployment, test plan

## Abstract

The targeted shortening of sensor development requires short and convincing verification tests. The goal of the development of novel verification methods is to avoid or reduce an excessive amount of testing and identify tests that guarantee that the assumed failure will not happen in practice. In this paper, a method is presented that results in the test loads of such a verification. The method starts with the identification of the requirements for the product related to robustness using the precise descriptions of those use case scenarios in which the product is assumed to be working. Based on the logic of the Quality Function Deployment (QFD) method, a step-by-step procedure has been developed to translate the robustness requirements through the change in design parameters, their causing phenomena, the physical quantities as causes of these phenomena, until the test loads of the verification. The developed method is applied to the test plan of an automotive sensor. The method is general and can be used for any parts of a vehicle, including mechanical, electrical and mechatronical ones, such as sensors and actuators. Nonetheless, the method is applicable in a much broader application area, even outside of the automotive industry.

## 1. Introduction

Nowadays, the steadily increasing efficiency of product development is a must. The need for new functionalities and new sensors is highly relevant, especially in the automotive industry [1]. In most cases customers require increased functionality of products and decreased cost, while reliability requirements remain the same or have been increased. Customers expect robust products, but usually do not accept higher prices. Robustness has become a profoundly desired and cited feature of products [2]. The need for robustness is also crucial in sensor development (see, e.g., [3]) where the challenges are related to the large degree of variation in design parameters [4,5,6].

Developing a robust product includes many—sometimes very sophisticated—activities, but the final step is always to verify the design and production [7,8]. Verification proves that the product—coming from serial production—will fulfil all the requirements its entire planned lifetime [9]. Requirements mean that the product must maintain its functionality within any realistic environment. For verification purposes these requirements have to be precisely formulated [10].

The long-requested lifetime of products sometimes results in long, time-consuming and expensive verification methods (which are disadvantageous for both the supplier and customer). Shorter but sophisticated indirect verification methods (e.g., accelerated tests) are sometimes hardly acceptable to customer due to their complexity and inherent degree of uncertainty [11]. Furthermore, part of the challenge is that existing tests and test equipment may unconsciously be biased towards their use in the verification process. Verification—based on habits or standards—may not prove the product’s robustness until the first real failure in the field occurs as unexpected failure modes sometimes happen in unexpected field conditions.

This situation highlights a need for an approach that simultaneously ensures that verified products will not fail in field conditions, cost-effective and can convince customers. The precise identification of the necessary and sufficient verification methods (test loads and measurements) is needed to fulfil this target, which also involves the precise identification of the targeted robustness.

In the development of autonomous vehicles, a “requirement-driven approach” is used to decrease the required resources as well as increase reliability [12], and “scenario-based testing” is utilised for the design of scenario-based tests [13].

Generally, neither the exact reproduction of the use conditions or the required time are feasible. That is why different verification methods/test are defined instead. ISO standards were established within the automotive sector to help find such adequate and acceptable verification methods: the ISO 16750 series (ISO 16750:1,2,3,4,5) “Road vehicles—Environmental conditions and testing for electrical and electronic equipment” gives general help specialized to automotive products, being thus rather adequate.

Nevertheless, even if the ISO 16750 series are relatively comprehensive and specialized expressly within the automotive, verified products sometimes fail in real use conditions. It can also happen that the verification process is too long, the loads are too excessive, or sometimes even different from the real use caseloads, which also indicates that the verification of robustness of a given product needs its own verification approach.

Despite all of the above approaches, which show that the engineering community has a shared understanding of robustness, there is a need for a systematic method that supports the verification of products regarding robustness.

The approach proposed in this work:

- is in accordance with everyday experience and expectations, namely a product is regarded—and verified by means of measurements—to be robust if it works under harsh conditions,
- derives the necessary verification activities (tests) by means of a step-by-step manner: from use case scenarios—through noises—through phenomena—to test loads,
- defines the measurability of robustness on a proportional scale.

The principle of the developed method is the following: Verifying the robustness of the product is equivalent, that the product maintains its functionality under the assumed conditions. Verifying that the product maintains its functionality under the assumed conditions is equivalent, that (verifying that) those changes, which can directly cause deviation from its intended functionality will not do this (if they are within a specific, realistic range). Verifying that these changes will not deviate the product from its intended functionality is equivalent, that (verifying that) their causes which are physical quantities will not result in these changes (if the amounts of these physical quantities are within a specific, realistic range). Finally, verifying that physical quantities will not result in the above (negative) changes is equivalent to the test loads that represent these physical quantities not resulting in the (negative) changes.

In this way, the noises, which cause the product to deviate from its intended functionality, are translated in a step-by-step manner into test loads. Passing the tests is equivalent to the fact that the product maintains its functionality under the assumed conditions, which results in the robustness of the product itself. Furthermore, if the product does not pass one or more tests, the robustness measure can be calculated, which is the ratio of passed tests to all tests. According to this, the proposed approach is the first to answer the question of how robust a product is. The exact answer to this question can help validation engineers in their work and in their communication with non-technical interested parties. The method can be part of requirement and system engineering activities, which target the robustness of a product. In part of the negotiation, it can be used in the quotation phase with the customer to gain confidence.

Based on the above considerations, the proposed method starts with defining the requirements for the product related to robustness, before these requirements are translated into test loads of the verification in a step-by-step manner.

Following this concept, the structure and main contributions of the article are as follows:

- A novel definition of robustness is proposed in Section 2.1.
- The developed method is based on the logic of Quality Function Deployment (QFD). In Section 2.2, the method that uses several Houses of Noises to derive the final test loads from a detailed description of the use case scenarios is presented in detail.
- In Section 2.3, the relationship of the method with regard to the widely used Parameter Diagram is pointed out. The importance of the quantification of the test load is discussed. Furthermore, it is presented that adequate quantification is necessary for both the effectiveness and cost-effectiveness of the verification.
- In Section 3, the method is explained through the development of an automotive sensor (Section 3.1); moreover, some tricks and possible pitfalls are pointed out (Section 3.2).
- Finally, in Section 4, conclusions are drawn, pointing out the benefits of the method.

## 2. Scenario-Based Noise Deployment

In this Section, the developed method, Scenario-based Noise Deployment (SND), is discussed in detail. The relationship between Quality Function Deployment (QFD) and the formalization of the method is introduced.

### 2.1. Definition of Robustness

Robustness itself is a popular desired and warmly welcomed feature of modern-day products in several industries and product segments and highly relevant in sensor design [14,15]. Many scientific articles deal with the robustness of a specific product, but considerably fewer with robustness as a general problem [2]. In the following overview, the most commonly used definitions of robustness are presented. The most famous definition is from Taguchi [16]. “Robustness is the state where the technology, product, or process performance is minimally sensitive to factors causing variability (either in the manufacturing or user’s environment) and ageing at the lowest unit manufacturing cost”. Another essential definition reads: “Robust design is defined, as the design that satisfies the functional requirements even though the design parameters and the process variables have large tolerances for ease of manufacturing and assembly. This definition of robust design states that the information content is minimized” [17]. As the authors emphasize, “Although different expressions are used, their meanings are similar. A common aspect of the definitions is that robust design is a design insensitive to variations, and this concept is accepted in the engineering community [2]”.

The reliability of products should be considered with regard to failure avoidance rather the probability of failure. From this viewpoint, the designers should focus on the critical product functions. Making the design insensitive to unavoidable variations is called robust design that can be supported by Variation Mode and Effect Analysis (VMEA) [18], which technique is similar to Failure Mode and Effect Analysis (FMEA), which is widely used in sensor development [19] and risk evaluation of vehicle failure modes [20]. Robustness was similarly defined as “the variance of product performance due to environmental noise” [21], and a similar approach pointed out the basis of the concept of Reliable Design Space (RDS), within which any design satisfies the reliability requirements [22]. Total robustness itself together with its definition is a significantly timely topic. Meanwhile, the measurability of robustness seems to be less researched. Nevertheless, a few papers on this have been published recently [23,24].

Before giving a new definition of robustness, it is worth collecting the possible requirements for this definition. Even if it is a natural expectation that the definition is exact and precise, its usability must also be acquired. It would not be very meaningful to set a definition that would not help us achieve the real goal: the robustness of the products themselves.

A definition is needed, which will:

- help the user to decide whether a given product can be regarded as robust or not,
- quantify the robustness of a given product,
- help the user to verify the robustness of a given product,
- be suitable to rank products (from a robustness point of view) which belong to the same class at least on an ordered scale but ideally on a proportional scale as well,
- be as general as possible.

The proposed definition of robustness: A product is robust (in a given environment) if (and only if) the following statements are true. The product maintains its intended functions and features in the presence of assumed noises which are present in the assumed use case scenarios. Robustness is defined by the ratio of good cases to all cases if these cases cover all possible scenarios and reflect the relative importance of the error-free scenarios vs. all scenarios.

The typical sensor performance characteristics are: range, linearity, sensitivity, resolution, response time, precision, hysteresis and stability [25]. The robustness of sensors can be evaluated for each of these characteristics by analysing that the the characteristics of the sensor are in the specified range in the presence of the assumed noises which are present in the assumed use case scenarios.

The above definition of robustness provides the basis for the developed method presented in the following subsection.

### 2.2. Details of the Method of Scenario-Based Noise Deployment

**Figure 1:**
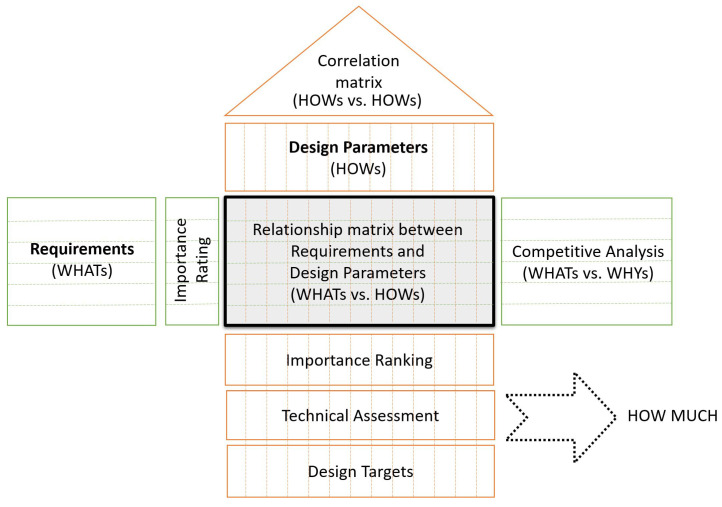
Basic QFD structure or “The House of Quality” based on [29].

The developed method, SND, is based on the logic of QFD. QFD is a widely known and preferred approach in quality management, used to apply customer expectations to engineering solutions. Even though it was invented in the late 1960s by Professors Shigeru Mizuno and Yoji Akao [26], over the last decade, many reviews have been published about it [27,28]. The basic QFD structure is presented in Figure 1:

The proposed method starts by identifying the requirements regarding the robustness of the product, which involves describing the conditions the product is assumed to be able to withstand while maintaining full functionality (use case scenarios). The proposed method describes how the translation of these use cases into more exact physical phenomena and finally into measurable physical quantities as test loads is achieved. These test loads will characterize the use-case conditions with measurable physical quantities enabling the verification (either Design Verification or Production Verification) of the product. The method flow is most easily understood through the example that will be presented in Section 3.

Ideally, the amount of verification resources is proportional to the corresponding risks. A calculation, which finally provides a ranking of test loads proportional to the corresponding risk, is essential. In the proposed translation process—besides identifying the adequate subsequent terms in the corresponding House of Noises the correct quantification of these terms which expresses their relative importance is performed. The output of the method is the correctly ranked test loads together with their relative importance.

**Figure 2:**
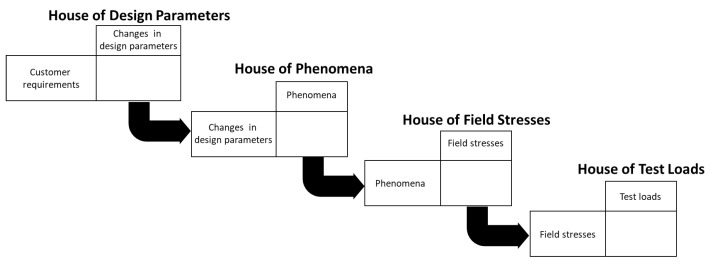
Cascade of Houses of the proposed Scenario-based Noise Deployment (SND) method.

The proposed method is schematically presented as the cascade of four Houses in Figure 2. In Figure 2, the logic of the method flow is shown. The robustness-related requirements are defined at the beginning of the process as effect-cause relationships with direct influencing factors. These influencing factors include technical system characteristics, design specifications (e.g., accuracy), system usage, and environmental impacts. The influencing factors are usually also in effect and cause the relationship with each other. The main benefit of the proposed method is that it supports structuring the factors in different House of Noises. The first House, the House of Design Parameters, connects the robustness requirements and their direct causes, the change of design parameters. The second House, the House of Phenomena connects the outcome of the House of Design Parameters to their direct causes. The third House, House of Field Stresses, connects the outcome of the House of Phenomena to their direct causes. In all the houses, the rows and columns are in a causal relationship until the final House, the House of Test Loads, just like in QFD. According to this logic, the method is based on the cause–effect analysis of the influencing factors and structures them to ensure that the houses represent as direct influences as possible.

The deployment logic can be seen in Table 1, which shows in detail how the House of Noises processes inputs from the previous House of Noises into outputs.

The last House of Noises is the one with columns that only contain loads as controllable physical/chemical quantities.

The advantages and the relationship with QFD of the developed method are presented in detail in the following.

While the precise description of the conditions of use—especially with regard to the physical terms of the stresses—seems to often be difficult, the description of the conditions of use in the language of the customer seems to be quite natural, just like in the case of the customer requirements in QFD.

What is common in the QFD approaches is “translating the voice of customers into technical languages” [28]. Nevertheless—even if the voice of the customer can and theoretically does cover all the customer’s expectations, including latent, unspoken expectations as well—considerably less attention has been paid to the conditions the product is supposed to work in. It can be said that targeting robustness means that much more attention has to be paid to these conditions since if they fall outside of the operating range of the product, the (expressed) expected functions of the product will be weakened or lost. What is problematic is that while the customer describes the above condition (customer usage) in their own language (if at all), the physical or chemical phenomena, which directly cause the failure or weakening of its function, should be characterised as physical quantities. It is worth noting, that both customer usage and environmental factors—as usually formulated with physical quantities—are incorporated into the Parameter Diagram (for more details, see Section 2.3), but without identifying of their exact relationship.

In SND, the conditions described in the “language” of the “customer” have to be translated into the “language” of physical/chemical quantities. On the other hand this language of the customer uses the words and style of requirements engineering but on the other hand, describes the situations the product is assumed to encounter when in use.

The other “end” of the translation is the aforementioned physical/chemical quantities, the amounts of which can quantitatively describe the phenomena which directly cause the failures or weakening of the functions. The following example illustrates this concept: if how the physical touch of the user can (negatively) change the quality of the surface of the handle of a tool (situation: the language of the customer) is of concern, and it is known that the handle—which is made of metal—can corrode, quantities of carbamide, NaCl, KCl and lactic acid (from the wet hands of the customer may be determined), the amounts of which influence corrosion most substantially.

A detailed example will be presented in the next section.

**Figure 3:**
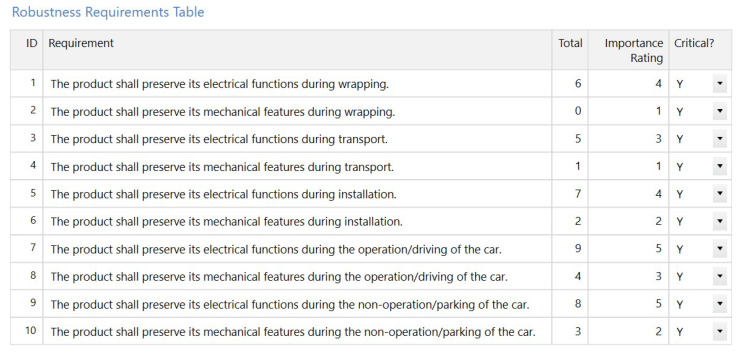
The formulation of requirements regarding robustness. It can be seen that robustness means that the product preserves its functions and features during different conditions.

In Figure 3, the requirements regarding robustness can be seen. All the requirements which define what we mean on robustness can be seen here. The requirements have initial importance ratings, which can be gained with any pair-wise comparison methods. It is important that the rating of the requirements shall be on a proportional scale, resulting in a specific rating of requirements compared to the sum of the rating, which will show the contribution of that requirements. Since the calculation in further steps in the House of Noises also has this feature, eventually, the ratings of the final tests will also be proportional.

**Figure 4:**
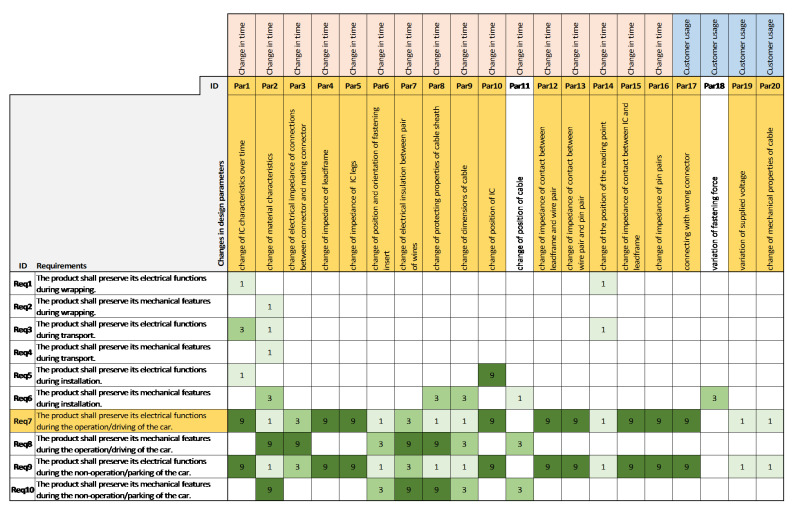
House of Design Parameters is the first House of Noises, which contains the requirements vs. changes in design parameters. The House of Design Parameters identify the direct causes, which result in the failure of the product. These direct causes are physical or chemical phenomena.

An example of such a results table is presented in Figure 4. In the rows of this table, the requirements of the product, and in the columns, the physical phenomena, can be seen, which are in direct relationship with the requirements: “the product shall preserve its mechanical features....”. One corresponding phenomenon (which hinders this requirement) is “change of material characteristics”. The numbers show the strength of the relationship, 9, 3, and 1 denote a strong, medium and weak relationship, respectively—similar to a typical House of Qualities [30].

The distribution of the responsibility for the robustness is calculated similarly to that of the QFD. When initially applying the method, the product’s robustness requirements are defined and the importance of every requirement is determined. This importance of every requirement—which account for the product’s robustness—is deployed and distributed among the test loads. After deployment, every test load accounts for a certain amount of responsibility for the robustness of the product. First, the importance is defined for each requirement, where the sum of the degrees of importance must be equal to 100 (Equation (3), regardless of the number of requirements. It is important to declare that by the importance, the contribution of the given requirement to the “whole” is meant, namely towards the product’s robustness. The relative importance of the requirement for robustness is calculated by a pairwise comparison using the Analytic Hierarchy Process [31]. After the robustness has been distributed among the requirements, further calculations follow the method described in [32].

The results of the evaluations are presented in four tables represented by four matrices $A_{i},i=1,\ldots ,4$, where i denotes the Houses as follows: i=1 represents the House of Design Parameters, i=2 the House of Phenomena, i=3 the House of Field Stresses and i=4 the House of Test Loads.

The input of the analysis is the importance of the requirements, which are found in the rows of the House of Design Parameters: $u_{1}={\left[u_{i,1},\ldots ,u_{i,n_{1}}\right]}^{T}$, where $n_{1}$ denotes the number of the rows in the House of Design Parameters. The results of the House of Design Parameters can be calculated by Equation (1):

(1) $$y_{1}={\left(u_{1}\right)}^{T}A_{1},$$

where $y_{1}$ denotes the vector of the results of the House of Design Parameters.

The end result of the method can be calculated by Equation (2):

(2) $$y_{4}={\left(u_{1}\right)}^{T}A_{1}A_{2}A_{3}A_{4}.$$

Equation (3) yields the importance of each test loads:

(3) $$u_{i+1}=y_{i}/\sum_{j=1}^{n_{i}}y_{i,j}\cdot 100.$$

**Figure 5:**
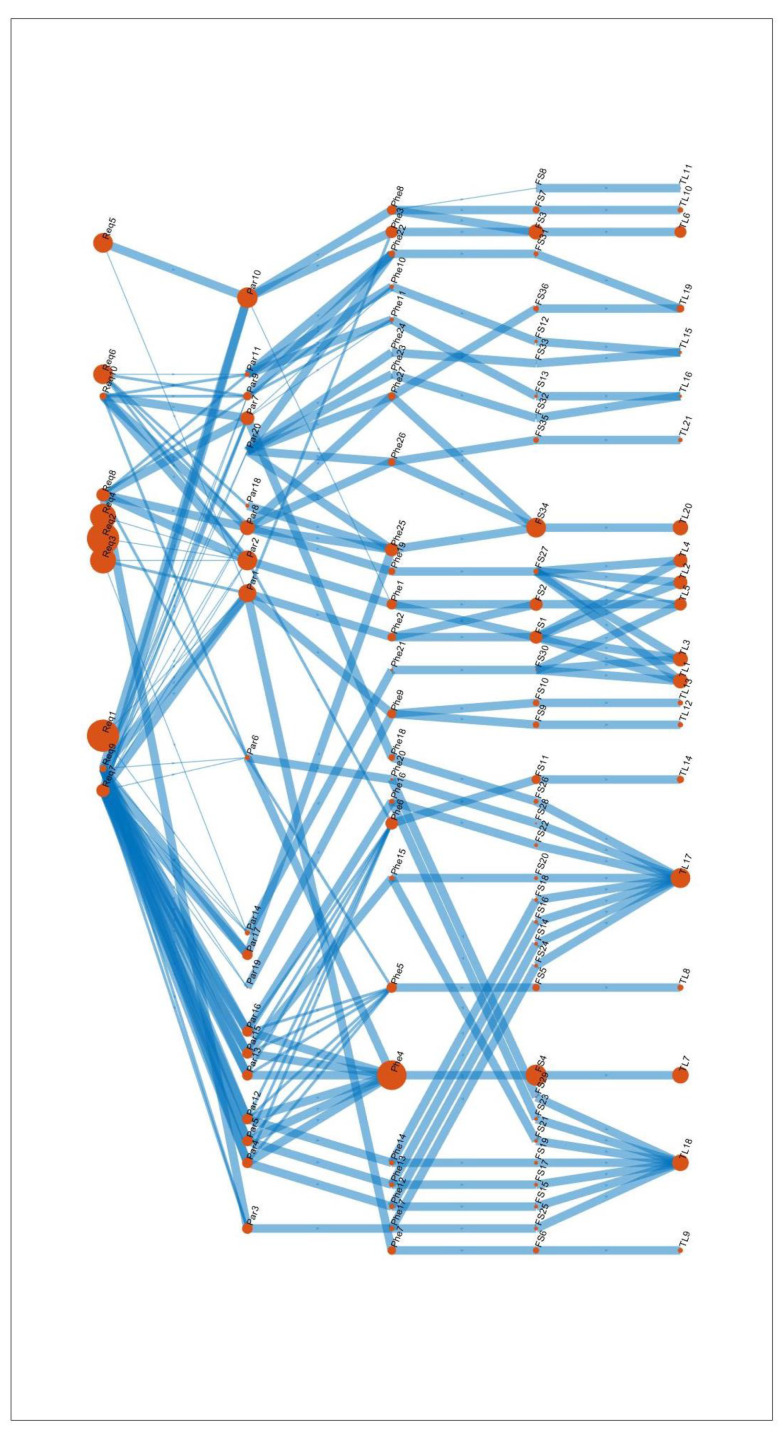
The network of connections between the Houses. The size of the red dots symbolizes the importance of the specific test loads, field stresses, phenomena, design parameters and requirements. The thickness of the lines represents the strength of assignments between each House.

The method can be easily visualized as a network. In Figure 5, the network of connections between the Houses is represented. The size of red dots symbolizes the importance of the specific test loads, field stresses, phenomena, design parameters and requirements. In the last row, which is the output of the House of Test Loads, the size of the red dots is parallel to the rank of test loads. The thickness of the lines represents the strength of assignments between each House.

The proposed network representation is beneficial as it supports the visualisation of how the requirements are propagated through the houses and allows the utilisation of the tools of network science. For example, the most influencing elements of the network can be calculated by the betweenness centrality measures that reflect how many (shortest) paths go through a given node or edge. Such analysis can be helpful for sensitivity analysis and identifying the bottlenecks of the test plans.

### 2.3. Relation to Other Methodologies

**Figure 6:**
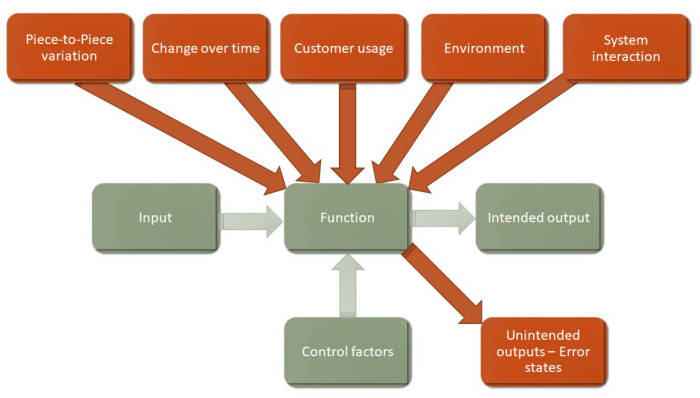
P-Diagram showing noises that may cause the failure modes of the function of a product.

Parameter Diagrams (P-Diagrams) is a widespread technique in the automotive industry to determine robustness [33]. A P-Diagram defines five categories of noise as the potential causes of error states: change over time, customer usage, environmental conditions and system interactions as field noises, and piece-to-piece variation as manufacturing noises (see Figure 6).

On the one hand a P-Diagram is easy to understand and use to identify the most important noises as the causes of the error states. On the other hand, it should be noted that the categories (and the items in the categories) do not realize an orthogonal space with regard to the causes of the error states. Change over time contains physical or chemical phenomena which lead to the given failure mode, but are usually not direct causes of the error. Environmental conditions contain the description of the environment, but likewise are usually not direct causes of the error. Customer usage used to be a well-described activity of the customer, which may (potentially) lead to a certain failure, but is also usually not a direct cause of the concerned failure modes. The piece-to-piece variation (manufacturing variation of critical design parameters) is also an important cause of error states as it has significance in the so-called PV (Production Validation). In contrast to Design Validation (DV), the test targets to verify that the parts from the serial production also comply with robustness requirements. Correct production validation also requires proper sampling, making the whole validation process even more complex.

**Figure 7:**
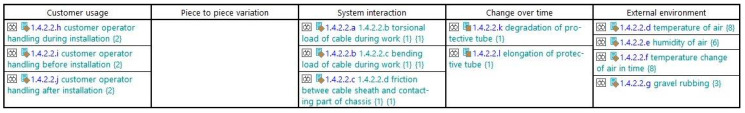
The P-Diagram that is used for classifying the noises.

The P-Diagram is undoubtedly a useful tool which helps to identify the root causes of the error states within the noises, but is noticeably an “offline” tool that is not fully integrated into the data flow. Even if currently used Failure Modes and Effects Analysis (FMEA) software like APIS IQ-RM integrates P-Diagrams (Figure 7) with possible (cause-and-effect) relationships between noises and error states, no hint about the interrelationships between the noise categories is given suggesting that they are all independent causes of the concerned error states.

SND utilizes P-Diagrams that is, uses all of the noises which could have been identified by P-Diagrams, but goes further and places all these noises in a correct cause-and-effect relationship. SND builds on P-Diagrams and places this well-known tool into a comprehensive method flow, which finally yields a tactile result: a test plan for robustness.

The variation in the design parameters is part of the category in the P-Diagram named “piece-to-piece variation”. Besides this variation—which is a variation between pieces—another change in the design parameters is present, namely change over time, which is another category in the P-Diagram as well as a noise category to choose from.

These noise categories in the P-Diagram significantly help to identify the direct causes of luck of robustness. On the other hand, belonging to a specific noise category does not mean that it is a direct or indirect cause of luck of robustness. This is a case-by-case situation and a P-Diagram simply helps to find noises seeking them in the given noise categories.

SND utilizes the thinking of P-Diagrams while also taking into consideration the cause and effect relationships between the noises in P-Diagram. The structure of the House of Noises preserves and visually presents these cause and effect relationships. The additional benefit of using House of Noises is that it is simple, friendly and similar to the House of Qualities and hence makes the approach applicable to a wide range of users.

Naturally, QFD is not the only method that can be used to structure design concepts. Its most relevant alternatives are the Pugh or Decision Matrix [34], the Kano Model [35], the Axiomatic Design (AD) [36,37] and the Design Structure Matrix (DSM) [38,39]. The Pugh Matrix is a criteria-based decision matrix that uses criteria scoring to determine which of the several potential solutions or alternatives should be selected. The Kano model of customer satisfaction identifies which function or requirement of a product or service achieves proportionate customer satisfaction [40]. The Kano model of customer satisfaction also determines which component of the system does not bring satisfaction or value to the consumer, but the lack of this component will cause dissatisfaction to the consumer [35]. In AD, two axioms provide a solid foundation for design teams to formalize design problems, conceptualize solution alternatives, eliminate bad design ideas, select the best of the proposed designs, and improve existing designs [36]. As a structured modeling method, the DSM is nowadays considered a good basis for system design. DSM is a good tool for mapping information flow and its effects on product development processes. It also visually represents the network of interactions between development activities or design goals and facilitates the analysis of these interactions [36]. A detailed comparison of these techniques and the benefits of QFD can be found in [41]. The detailed comparison of these techniques highlights that the quality of houses is an ideal framework for structuring the requirements according. The method also supports the quantitative analysis and provides tools for interpretable prioritisation of complex decision problems, like analysing a test plan according to the robustness of a product.

## 3. Application to Sensor Development

The example below shows the implementation of the aforementioned principles in the case of a sensor used in an automotive application.

### 3.1. Description of the Example

The deployment of the noises starts with the description of the scenarios using ordinary language as well as the terms and formulations of requirements engineering. Such sentences like “the product shall preserve its electrical functions during the operation/driving of the car” clearly describe the scenario or situation in which the product will likely be during its lifetime and are also suitable to be translated into more exact terms. As can be seen in the example, the House of Design Parameters (Figure 4) mainly contains changes, since the original requirement is translated into this sentence: “The product shall preserve its electrical functions despite the change of IC characteristic over time, despite the change of material characteristics, despite the change of lead frame characteristics” and so on. These are highlighted in yellow in Figure 4.

It can be seen that in the House of Design Parameters (Figure 4), all factors are either deviations over time (of the same pieces from an acceptable starting value) or between some pieces (which are considerably different from the average). According to logic (and experience), problems (in the case of a faultless design) arise from deviations from an ideal case.

It is also worth noting that these changes belong to either the (characteristics) of the product itself or the immediate environment, which is quite logical. As a result, the further deployment of noises is quite simple: the direct cause of these changes and later the direct causes of the aforementioned direct causes.

**Figure 8:**
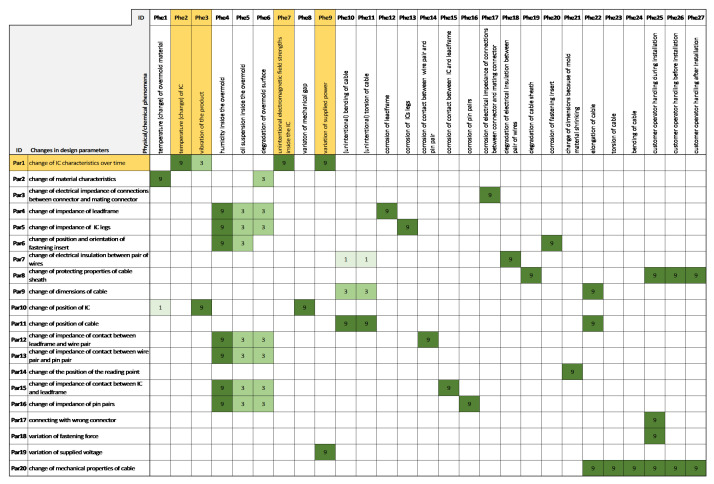
House of Phenomena: this is the second House of Noises: Changes in design parameters vs. Physical/chemical phenomena. The House of Phenomena connects the direct causes from the House of Design Parameters (output of the House of Design Parameters) to its direct causes.

In the following House of Noises, it can be seen how the translation is continued. The House of Phenomena (Figure 8) shows the direct relationship between the output of the House of Design Parameters and its direct causes.

**Figure 9:**
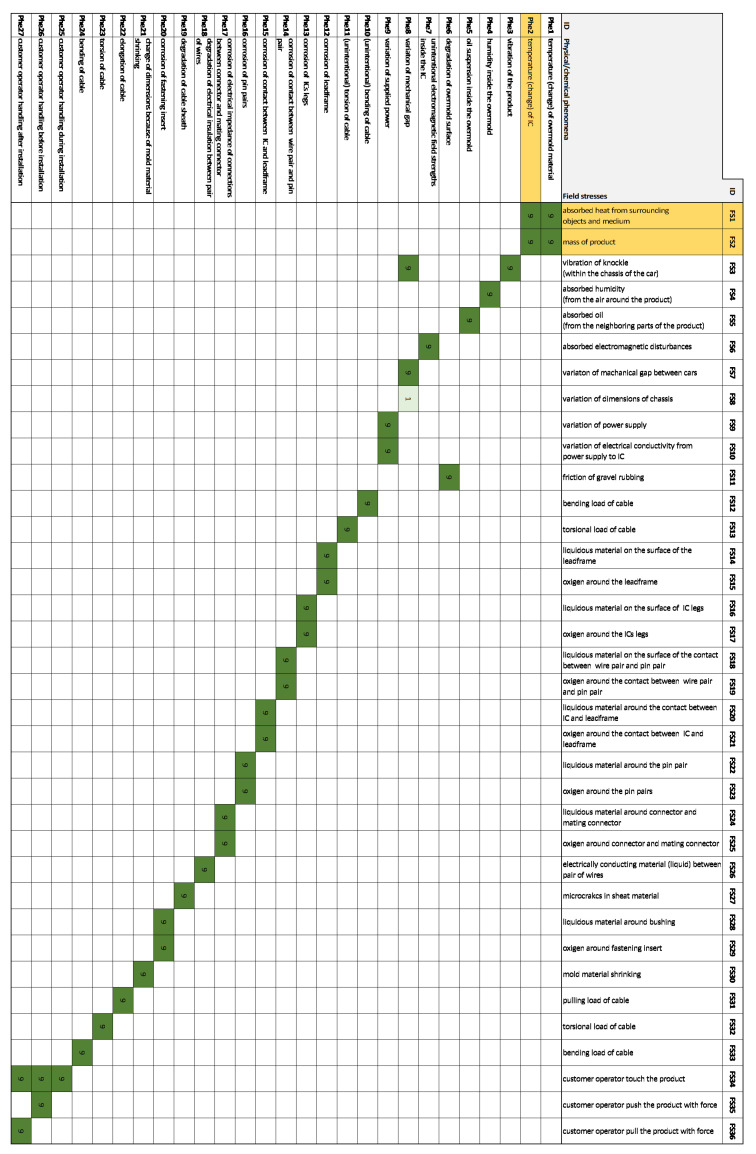
House of Field Stresses: this is the third House of Noises: Physical/chemical phenomena vs. Field stresses. The House of Field Stresses connects the direct causes from the House of Phenomena (output of the House of Phenomena) to its direct causes.

As an example, the “change of IC characteristic over time”—as a result of the first column—depends on the “temperature (change) of IC”, “vibration of the product”, “unintentional electromagnetic field strength inside the IC”, and “variation of the supplied power”. The number “9” shows that there are strong correlations between “change of IC characteristic over time” and the corresponding column factor. These examples are highlighted in yellow in Figure 8. In the next step, the causes of these factors will be once more identified, this will be the House of Field Stresses (Figure 9).

**Figure 10:**
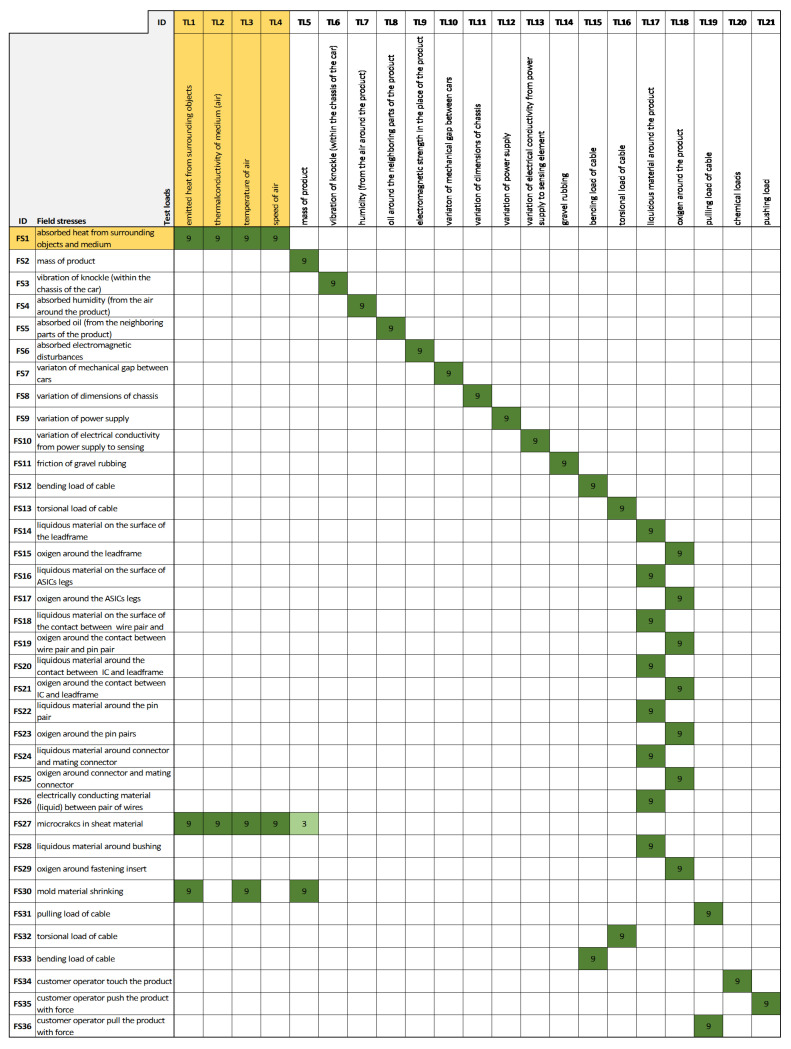
House of Test Loads: this is the fourth House of Noises: Field stresses vs. Test loads. The House of Test Loads connects the direct causes from the House of Field Stresses (output of the House of Field Stresses) to its direct causes. In the example, this is the last House of Noises, which—as the output—contains the ranked test loads.

Following the aforementioned example, now the “temperature (change) of IC”, “vibration of the product”, “unintentional electromagnetic field strength inside the IC”, and “variation of the supplied power” are further deployed with regard to their causes. By now choosing “temperature (change) of IC”, the corresponding causes of this are “absorbed heat from surrounding medium” and “mass of product”. From then on—in the next House of Noises, namely the House of Test Loads (Figure 10)—the causes of these factors are identified. These are highlighted in yellow in Figure 9.

By continuing the example and now choosing “absorbed heat from surrounding medium” as the consequence in the House of Test Loads, it can be seen that the causes of this factor are “emitted heat from surrounding objects”, “thermal conductivity of medium”, “temperature of air” and “speed of air”. These are highlighted in yellow in Figure 10 and are already controllable physical loads that the (test) engineer can set to their values. Since the values of all these loads are obtained by calculating the QFD, their values set up a proportional ranking according to their contributions to the robustness of the product (supposing the success in the test).

In general, the number of Houses of Noises is not determined in advance, the concrete situation defines how many are needed. The final goal is to achieve the test loads, which represent those stresses the product is assumed to be able to withstand under field conditions. By achieving these test loads with their corresponding ranking, the basis for the verification of the robustness is obtained.

### 3.2. Discussion of the Applicability of the Method

Although the logic from that point is relatively straightforward, there may be some pitfalls that can be avoided by persistent strict logic and the necessary knowledge of the physics of the related phenomena. One good example is when the “temperature change of the overmolded material” is identified as what can cause a problem and the direct logical (and physical) cause is the “absorbed heat” by this material. The next logical step is to identify the potential sources of this heat and all the other influencing factors like the surrounding medium (air), speed of the medium, distance of the product from heat sources and the product’s mass.

It can also be realized that the method works only if adequate knowledge of the related phenomenon is available and used. For example, when looking for the cause of the “degradation of cable sheaths”, it is known that our everyday knowledge can be obtained from different kinds of loads like bending and torsional ones. Nevertheless, the direct cause of degradation is—in a logical sense—“between” the degradation phenomena and these loads, that is, “micro-cracks in the sheath material”, moreover, the cause of these cracks is only obtained in the next House.

It is worth looking at the visual pattern of the Houses (the analysis of which is highly supported by the proposed network representation). Just like in translation in different languages, a one-to-one translation between terms would be ideal, but not always possible. One-to-one translations between consequences and direct causes would result in a diagonal pattern, so any severe deviations from this diagonal pattern would draw our attention to something important or at least interesting.

If a consequence has too many direct causes, this may point out that the consequence is formulated too generally. In the example, this was the case initially with the term “change of mechanical properties of cable”. By perceiving that this term has too many causes, it may be understood that simply referring to “mechanical properties” is too general. Even if there is a chance that later on this will be corrected by splitting the reasons adequately into more concrete causes, it would be better to correct the situation by “here and now” and identify the precise material properties themselves.

Even if it is evident that the quantitative description of these factors may sometimes (often) need a tremendous effort that may not be worth it or even impossible, identifying these factors just needs consistent thinking. This is an essential practical viewpoint since verification can fall mainly because of two reasons: the extent (including the time) of the test loads does not represent the real field stresses, or adequate stresses are only absent from the test loads because they were not identified.

Such a question is related to accelerated tests. Establishing the right quantitative model between field stress and test stress can be challenging in some cases and may require many experiments. Using a less-than-optimal model, however, only results in either too early a failure or too excessive test loads. On the other hand, entirely neglecting a field noise and not representing the test loads can lead to a failure caused by “unknown” noise. This latter situation is handled by the method, which only needs the contributions of sufficiently qualified experts.

It is interesting to see that from House to House the “translation” tends to become a word-by-word translation or sometimes just repeats the same words, that is, less and less “translation” is needed. The deployment that is the “translation” is complete if all terms in the columns are controllable physical quantities, which can be set during verification. This regularity is also recognizable in terms of the diagonal geometric pattern, which is finally formed.

It is also instructive to see some examples that show that despite all structured method flows and rules, common sense, some knowledge and critical thinking are still needed to end up with beneficial solutions. Such an example is the “absorbed heat from surrounding objects and medium” evaluation which, on the one hand, is not a clearly defined quantity, but on the other hand, cannot simply be substituted with a defined amount of heat, since this—at the time of this deployment—is usually unknown. Nevertheless, it provides an input for defining the corresponding test loads at least qualitatively, which is important for the verification.

What matters in total is that all the derived test loads are feasible, realizable and—altogether—represent the corresponding scenarios and ratings as well as show their relative importance. The relative importance of the test loads is more important than a precise mathematical function between a given field noise and the corresponding test load since in the case of non-significant field noises, it is not worth seeking the exact relationship between a field noise and test load.

Tests—at least some of them—are either expensive or time-consuming or even both. It seems evident that the efforts made to carry out a test need somehow to be proportional to the risk that should be avoided. Therefore, the quantification of the relationship between the risks (result from the field noises) at the end of the noise deployment is inevitable. The relationships between the importance of these test loads must also be defined and quantified besides the definitions of the test loads themselves.

**Figure 11:**
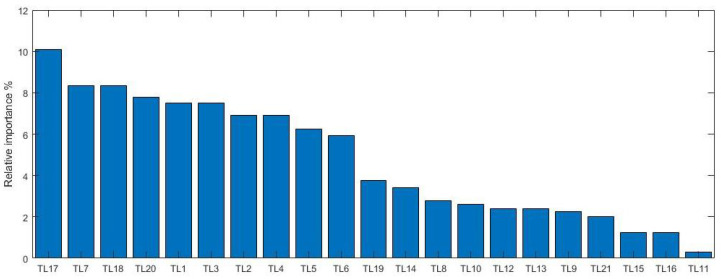
The ranking of the test loads after the last House of Noises. Given the method, the ranking yields a proportional scale: the importance of a successful test from a robustness point of view is proportional to the resultant value.

In the following example, the test loads along with their ranking can be seen in Figure 11:

By evaluating Figure 11, at first, what can be seen seems quite usual: different test loads have different orders of importance. However, if how much time and how many resources a concrete test needs to be successful are also considered, it is possible to focus our attention on crucial goals. If—for example—passing the “TL19” test currently accounts for 20% of the test cost, it is worth considering changing the test requirements themselves: after all, the goal of our customer and us is the robustness of the product and not successful tests.

By expounding on the details and taking the top three test loads, namely TL17, TL7 and TL18 (which are humidity of the air around the product, liquid material around the product and oxygen around the product, respectively, in the concrete example) into consideration, as well as following the deployment backwards until the requirements these test loads are derived from results in the most important and most highly ranked requirements themselves (req7, req8, req9 and req10). By looking at their content, it can be seen that these requirements define that the product should “work” during the operation of the vehicle and, therefore, these are much more important (during its operation, these have safety aspects) than all the others (with only economic aspects). This also points out how the method flow set up the ranking of test loads, which—in their order—map the importance of the robustness requirements.

Qualitatively this ranking shows us those test loads in the right order, which—if they applied to the product—would correspond to the most important and most avoidable failure mode. In other words, this means that if all of these test loads are applied to the product and no failure modes occur, the product can be regarded as robust —because of the noise deployment flow—for the defined scenarios described at the beginning of the deployment.

Ranking of test loads is, however, insufficient to describe the relationship between test loads. Even if the ranking is good, the right order of the test loads (from a risk point of view) does not show how much cost it is worth spending to avoid the corresponding failure mode. In other words, the relative importance of the test loads is equal to the proportion of the worthy costs of these tests to be spent to avoid the corresponding failure mode.

The proposed method was compared to the most widely used design support techniques presented in Section 2.3. We concluded that the QFD is the best among these techniques as it structures the robustness requirements in the most interpretable manner. We also generated the P-Diagram for classifying the noises (some of the related results are presented in Figure 7. Our experience related to the generation of the P-Diagram showed that the P-diagram does not present cause-and-effect relationships among the studied noises, which can provide difficulties at evaluating their importance.

The result of the analysis can be validated based on failure data from the field. Unfortunately, in the automotive industry, the intended lifetime is around 15 years, and naturally, at the design phase of the test plan, we do not have such information. Nevertheless, the method can be falsified if field failure data contradicts the (usually zero) predicted defects within the monitored warranty time. As such information is not available at the design of a new product/test plan, the results can be quantitatively or qualitatively validated by experts knowledge and data coming from similar problems. The proposed technique was proven applicable and promising to provide a realistic result with a transparent and well-structured form based on this validation process.

## 4. Conclusions

In this paper, a new verification approach is presented, which—utilizing the logic of QFD—uses Houses of Noises to derive the necessary test loads representing the customer usage of a product.

The first step of the developed method is to describe the requirements regarding the product’s robustness, namely to describe the conditions the product is supposed to be able to withstand while maintaining full functionality. With the proposed method, the use case scenarios are translated first into exact physical phenomena and finally into measurable physical quantities which can be applied as test loads enabling the verification (either Design Verification or Production Verification) of the product.

The logic and calculation of the method flow assure that the final ranking of the test loads is proportional to the product’s robustness. Every test load is characterized as a percentage equal to the percentage of the product’s robustness if the product passes the test. In case the product fails some tests, which are—hopefully—among the least important ones, it is still possible to state the extent of the product’s robustness in the form of the ratio of the aforementioned percentage of the passed tests. In a product development process, the development time is crucial and finding an acceptable level of robustness within the shortest development time is invaluable.

This method makes it possible to focus on the critical robustness goals and help achieve either the desired robust product or make a demonstrably risk-based compromise. A significant benefit of the method is that if the product fails one or several tests, the resulting robustness is still interpretable and will be a quantity between 0% and 100%. A significant advantage is that it can create a well-founded deviation request to the customer and establish a risk-based compromise between different interests like the robustness of the product and the corresponding development time as well as development costs. Altogether, this can shorten the product development process while maintaining an acceptable level of safety and reliability of the product.

## Figures and Tables

**Table 1:** The inputs and outputs of the proposed SND method which show the logic of the relationship between the Houses of Noises.

	Input	Output
**House of Design Parameters**	Requirements	Changes in design parameters
**House of Phenomena**	Changes in design parameters	Physical/chemical phenomena
**House of Field Stresses**	Physical/chemical phenomena	Field stresses
**House of Test Loads**	Field stresses	Test loads
